# Cancer in female firefighters: The clinicobiological, psychological, and social perspectives

**DOI:** 10.3389/fpubh.2023.1126066

**Published:** 2023-04-12

**Authors:** Kenneth Robert Kunz, Kate Turcotte, Samantha Pawer, Alex Zheng, Amanat Purewal, Alyssa Wellar, Shazya Karmali, Len Garis, Larry S. Thomas, Ian Pike

**Affiliations:** ^1^Medical Oncology, Molecular Pharmacology, Victoria, BC, Canada; ^2^BC Injury Research and Prevention Unit, British Columbia Children's Hospital Research Institute, Vancouver, BC, Canada; ^3^School of Culture, Media, and Society, University of the Fraser Valley, Abbotsford, BC, Canada; ^4^Surrey Fire Service, City of Surrey, Surrey, BC, Canada; ^5^Department of Pediatrics, The University of British Columbia, Vancouver, BC, Canada

**Keywords:** women’s health, firefighters, cancer, prevention, policy

## Abstract

**Objectives:**

This study explored how demographic characteristics, life experiences, and firefighting exposures have an impact on cancer among female firefighters, and described the types and biologic characteristics of cancers as reported by women in the fire service.

**Methods:**

The online survey was available from June 2019 to July 2020. Questions related to demographic characteristics, lifestyle factors, firefighting exposures, and cancer diagnoses. Descriptive analyses characterized variables by the presence or absence of cancer. Qualitative data provided insight into both firefighting and cancer experiences among women.

**Results:**

There were 1,344 female firefighter respondents from 12 different countries, 256 of whom provided information on their cancer diagnosis. North American respondents made up 92% of the total. Those with cancer were older, had been in the fire service longer, had more career fires and toxic exposures, and were less likely to still be in active service. They also reported more tobacco use, and more full-term pregnancies. There were no differences in family history of cancer between the two groups. The average age at diagnosis was 39.0 years. The major types of cancer reported included breast (25.4%), cervical (21.1%), melanoma (20.7%), base cell/skin (16.4%), and uterine (14.8%). The cancer was detected when seeking medical attention for symptoms (42.1%), during routine health screening (29.8%), and during specific cancer screening (28.1%). The stage of cancer was reported by 44.5%, and 30.9% included the histopathological grade. Treatments included surgery (72.7%), chemotherapy (14.8%), radiotherapy (13.7%), and observation (13.7%). Challenges associated with cancer included psychosocial (33.2%), financial (18.8%), physical (6.6%), and spiritual (6.3%). Concerns about reporting a cancer experience to their employer included the desire to keep health information private (11.3%), a feeling of vulnerability (7.4%), and being perceived as weak (7.0%). Lack of support from their employer or insurer was also noted.

**Conclusion:**

Female firefighters experienced a wide variety of different types of cancers which may come earlier than similar cancers in the public. These findings can help inform resource allocation, the development of new policies, and the need for broader presumptive coverage to support female firefighters diagnosed with cancer.

## Introduction

Cancer is a principal source of chronic illness and one of the leading types of all-cause mortality in Western nations. In Canada, for example, cancer was the primary cause of death in 2019, representing 28% of all fatalities, followed by heart disease (19%) then unintentional injury (5%) (1). It is estimated that in 2022, more than 230,000 Canadians and 1.9 million Americans will receive a diagnosis of cancer (2, 3). Moreover, the relentless war being waged against cancer has seen trillions of dollars spent with the optimistic view that, one day, better diagnostic, therapeutic, and preventative strategies will stem this rising tide (4, 5). The net overall effect of these vast, expensive, and exhaustive efforts however has been that the cancer incidence and mortality rates have continued to inexorably rise.

Despite the determination of organized societies to reverse this trend, some purposeful and necessary human endeavors that are known to predispose to cancer, such as the myriad of duties and conditions associated with firefighting (6), must continue in their present forms relatively unchanged. In this light, numerous studies have confirmed an association between the discipline of firefighting and cancer. To date however, none of these investigations have included a large enough number of women to draw any specific qualitative or quantitative conclusions regarding the general health outcomes as they concern a diagnosis of cancer in a female firefighter. Parameters such as the types of cancers, the means of clinical presentation, the methods of diagnosis, the tumor stages and histopathologic grades, and the therapeutic strategies employed to help female firefighters are entities that remain largely undefined. An example of this absence of information is reflected in a 2006 meta-analysis combining 28 selected studies by LeMasters and coworkers, which reported on the cancer rates among a dataset of nearly 115,000 firefighters (7). Despite the statistical power of very large numbers, this investigation was only able to address cancer as it concerns male firefighters, as no information had been collected or was otherwise available on female firefighters. More recently, an expansive 2021 epidemiological pandect, encompassing 11 large systematic reviews on cancer incidence and mortality among many hundreds of thousands of firefighters, did not specifically address the health of female firefighters aside from citing one study on brain cancer which included a combined category of “males and females” in the inclusion criteria, but did not report out separately by sex (8).

There have been a handful of epidemiological reports dealing with cancer in male firefighters which have captured small subsets of women as an additional or incidental domain of inquiry (9–12). In these cases, numbers have again been too small to draw definitive conclusions except to say that, based on limited data, female firefighters have an increased overall risk for many types of cancers when compared to women in the general population. However, these studies did not have the capacity nor were they designed to consider the clinical and biological circumstances around the diagnosis, nor the social, psychological, financial, or career impacts that a diagnosis of cancer has on a woman in the fire service. This dearth of information was recently reemphasized by a series of focus group sessions involving 49 career women in the fire service (13). The report concluded that the risk of job-related cancer is a primary source of apprehension for these women and stressed the urgent and ongoing need to collect further information and to designate resources to address these deficiencies.

The lack information is understandable given the fact that, traditionally, relatively few women have been employed in the fire service. The International Association of Fire and Rescue Services has estimated that only about 9% of all firefighters worldwide are female (14). Although historically a male-dominated profession, new and emerging issues around workforce balance and equity is precipitating a shift in gender dynamics such that the ranks of female firefighters are now beginning to increase. In order to provide resources and support for this unique population, including expanding presumptive coverage for women firefighters, more actionable information is needed regarding the health and wellbeing of female firefighters, especially as it concerns a serious medical issue such as cancer, with all the personal and professional ramifications that attend such diagnosis.

An estimation of the magnitude of these concerns can be made by considering the current number of working female firefighters at elevated risk for cancer, and comparing it against the baseline cancer incidence and mortality rates among women in the general population. In the United States (US) alone, about 40% of all women will develop cancer at some point during their lifetimes, with 29% eventually succumbing to this disease (15). However, because of the well-known carcinogenicity of firefighting due to exposure to combustion products, building materials and flame retardants, firefighting chemicals, diesel exhaust, shift work, and more (6), researchers have identified a trend to higher cancer rates among female firefighters as compared to the general public. Daniels and coworkers reported that women in the fire service may have elevated cancer rates (Standardized Incidence Ratio (SIR) = 1.24, 95% CI 0.89–1.69) (10); while Ma et al. reported a significantly increased rate (SIR = 1.63, 95% CI 1.22–2.14) (9). Carrying this reasoning forward, if there are presently about 93,700 combined volunteer and career female firefighters in the US, it can be expected that many tens of thousands of these women will eventually go on to develop some form of cancer that has been sparked by their profession. Furthermore, because of the long latency periods (time between exposure and diagnosis) for some malignancies, many of these women may develop their illnesses years after they have retired from active service.

Although women share many biological similarities to men, the differences in reproductive anatomy and physiology determine that a diagnosis of cancer in a female firefighter will encompass a unique set of gender-specific problems. Women are vulnerable to many different forms of ‘female’ cancers which do not affect men, such as endometrial, cervical, vulvar, vaginal, ovarian, and fallopian tube cancers, in addition to up to 100 times more breast cancer than their male counterparts (16). Little is yet known about the range, severity, and burden of these female-specific illnesses; furthermore, such a list must also include the cancers that female firefighters are vulnerable to in common with their male colleagues, for example colorectal, bladder, and melanoma (8).

In terms of providing support for women at risk or those already diagnosed with cancer, more information and resource is needed for governmental regulatory bodies, insurance providers, and fire jurisdictions to meet the rising challenges around cancer prevention, education, or screening and surveillance strategies concerning cancer in female firefighters (17–20). Neither are there standardized and effective health policies across jurisdictions, nor sufficient resources allocated to initially aid and subsequently provide ongoing care for women in the fire service who have been confronted with a cancer diagnosis. One of the major barriers to collecting this information is simply being able to recruit a sufficient number of female firefighters who have been diagnosed with cancer, and who would further be willing to share these difficult personal experiences by participating in a lengthy survey. Our desire was to develop a method of appealing to and connecting with as many female firefighters as possible to report on these and related issues.

When studying fertility outcomes as they relate to maternal and child health among women in the fire service, Jahnke and coworkers solved this problem by designing and implementing an international, on-line, self-report survey with ‘snowball sampling’ techniques. From a relatively rare population, they were able to assemble a dataset of 1,821 female firefighters and determine that spontaneous miscarriage and delivery of low birth weight infants was statistically higher in this group and correlated with length of time in the fire service (21). The present study on cancer in female firefighters implemented a similar online survey, designed and interpreted by a medical oncologist (KRK), to gather information on the clinical, social, psychological, and financial ramifications that such an illness would encompass.

The purpose of this study was to describe the circumstances around a cancer diagnosis among female firefighters, with the following objectives:

1. Describe the types of cancers as reported by women in the fire service, including clinical stage and histopathological grade, how these cancers were detected and treated, the average age of onset, and length of occupational exposure-time.
2. Explore the types of work-related exposures and personal lifestyle factors that predispose toward a diagnosis of cancer.
3. Describe how being diagnosed with cancer affects the quality of life of a female firefighter.

## Methods

### Study design and recruitment

An online survey was developed to solicit self-reported information from women currently or previously working in the fire service in the United States, Canada, and elsewhere. From June 7, 2019 to July 19, 2020, prospective participants were directed to an online REDCap survey, available in both English and French, to learn more about the study, consent to participating, and complete the study survey as desired. Approval to conduct this study was provided by the University of British Columbia Children’s and Women’s Research Ethics Board (H18-03318). The survey was advertised on industry-specific magazine sites and blogs and via postings and email lists by agencies as described by Pawer et al. (22), and snowball sampling was employed (23). The purpose of the study was to describe work-related injury and cancer experiences among female firefighters.

### Survey content

Respondents were asked to provide information on their demographics, lifestyle (e.g., tobacco and alcohol use), firefighter career (e.g., department, rank), work-related injuries, and cancer and precancer diagnoses, as described by Pawer et al. (22). Other lifestyle information considered in this study include hormone use, experience of full-term pregnancy and delivery and if the respondent had breastfed, and family history of cancer. Respondents with cancer (either incipient or diagnosed) were asked to provide information on the mode of detection, stage and grade, and treatment received. Incipient cases were identified by survey respondents as “precancer” diagnoses. Participants were also asked to describe challenges related to their cancer, concerns about reporting their cancer, anything they would like the researchers to know about how cancer has affected their quality of life, and if they had anything to share about their career with the fire service.

### Data analysis

Descriptive analyses were conducted, with demographic characteristics, life experiences, and firefighting experiences described by the presence or absence of cancer/precancer. Cancer-specific data were further broken down by the type of cancer, mode of detection, stage, grade, and type of treatment received.

Continuous variables were presented as means and standard deviations if the distribution was normal and as medians and quartiles if the distribution was not, while categorical variables were displayed as counts with an associated proportion of the sample. Univariable comparisons were conducted between those who reported having cancer with the those who did not for each of the variables of interest to determine whether there were significant differences between the two groups. *T*-tests were used to compare continuous variables that were normally distributed, Wilcoxon rank-sum tests were used to those that were not normally distributed, and Chi-square tests were used to compare categorical variables. While the survey included a section about work-related injury, those analyses are presented by Pawer et al. (22).

Short answer responses were analyzed using inductive content analysis via NVivo 12 (24). Three researchers (SK, AP, AW) independently reviewed responses to identify common themes, which were then compared and discussed between the researchers to create final themes. Any discrepancies were discussed until consensus was reached.

## Results

### Demographics, lifestyle, and fire service experiences

A total of 1,344 female firefighters from 12 different countries responded to this survey, 256 (19.0%) of whom provided information on their cancer experience; either a diagnosis of incipient (precancer) or frankly invasive cancer. While nearly all of the respondents were born female and identified as female, there were a few cases of individuals born female and currently identifying as male or non-binary, and eight individuals born male and currently identifying as female (Table 1). Respondents were primarily located in North America, but also included those from Europe, Australia and New Zealand, and Asia. In total, 87.5% of respondents identified as ‘white’, with other ethnicities each accounting for fewer than 5% of the sample. The average age of respondents was 41.1 years (SD 10.1). Respondents diagnosed with cancer were significantly older than those without cancer (*p* < 0.001). There were no differences in height, weight, or body mass index (BMI) between respondents with and without cancer.

**Table 1:** Demographic characteristics of female firefighter survey respondents, by presence and absence of cancer.

Domain of inquiry	All respondents^1^ (*n* = 1,344)	Respondents without cancer (*n* = 1,067)	Respondents with cancer (*n* = 256)	Comparison
	Count (%)	Count (%)	Count (%)	*p*-value
Sex, Gender^2^				0.727^b^
Female, female	1,331 (99.0)	1,056 (99.0)	255 (99.6)	
Female, male	*	*	0	
Female, non-binary	*	*	0	
Male, female	8 (0.6)	6 (0.6)	*	
Location^3^				0.306^b^
North America	1,232 (91.7)	976 (91.5)	240 (93.8)	
Europe	62 (4.6)	48 (4.5)	11 (4.3)	
Australia and New Zealand	48 (3.6)	42 (3.9)	5 (2.0)	
Asia	*	*	0	
Ethnicity				
North America				0.552^b^
White	1,051 (78.2)	819 (76.8)	216 (84.4)	
Hispanic	55 (4.1)	46 (4.3)	7 (2.7)	
Aboriginal	43 (3.2)	37 (3.5)	6 (2.3)	
Asian	22 (1.6)	17 (1.6)	5 (2.0)	
Afro-North American	16 (1.2)	13 (1.2)	*	
Other^4^	114 (8.5)	101 (9.5)	13 (5.1)	
Europe				N/A
White	62 (4.6)	48 (4.5)	11 (4.3)	
Australia and New Zealand				0.733^b^
White	63 (4.7)	48 (4.5)	12 (4.7)	
Aboriginal	7 (0.5)	6 (0.6)	*	
Asian	*	*	0	
Other^5^	17 (1.3)	13 (1.2)	*	
Asia				N/A^b^
Asian	*	*	0	
	Mean (SD)	Mean (SD)	Mean (SD)	
Age (years)	41.1 (10.1)	39.6 (9.6)	44.2 (8.9)	**<0.001** ^a^
Height (cm)	168.5 (7.5)	168.6 (7.7)	168.4 (6.6)	0.769^a^
Weight (kg)	74.2 (14.8)	74.2 (14.4)	73.6 (12.6)	0.521^a^
BMI	26.2 (5.5)	26.2 (4.7)	25.9 (4.2)	0.554^a^

* = fewer than 5.Bold *p*-values indicate statistical significance.

1 Responses from respondents with or without a history of cancer and/or precancer may not add up to those of all respondents, as some respondents did not answer every question.

2 Sex is defined as male or female assigned at birth according to genetic characteristics; gender is defined as how individuals identify themselves in a social or cultural context.

3 North America (USA, Canada); Europe (UK, Ireland, Sweden, France, Germany, Belgium); Asia (China, Singapore).

4 Examples of other ethnicities include Middle Eastern and mixed ethnicities.

5 Examples of other ethnicities include mixed ethnicities.

a *T*-test.

b Chi-square test.

Respondents had an average of 14.5 years (SD 9.5) since starting in the fire service (Table 2). Those diagnosed with cancer were in the fire service for a significantly longer period of time as compared to those without cancer (*p* < 0.001). They also estimated experiencing a significantly higher number of career fires (*p* < 0.001) and toxic exposures (*p* < 0.001). Respondents with cancer were significantly less likely to still be in active service at the time of the survey (*p* < 0.001), having left due to health issues, a career change, or retirement. They were also significantly more likely to be career firefighters as opposed to volunteer or career/volunteer combined (*p* = 0.028).

**Table 2:** Firefighting experience and exposure history of female firefighter survey respondents, by presence and absence of cancer.

Domain of inquiry	All respondents^1^ (*n* = 1,344)	Respondents without cancer (*n* = 1,067)	Respondents with cancer (*n* = 256)	Comparison
	Mean (SD)/Median (Q1, Q3)	Mean (SD)/Median (Q1, Q3)	Mean (SD)/Median (Q1, Q3)	*p*-value
Years since starting in service	14.5 (9.5)	13.4 (9.2)	19.1 (9.4)	**<0.001** ^a^
Years since leaving service	5.3 (5.3)	4.4 (4.9)	6.0 (5.3)	0.196^a^
Estimated # career fires	40 (15, 150)	40 (15, 100)	100 (25, 250)	**<0.001** ^b^
Estimated # toxic exposures	5 (1, 25)	5 (1, 20)	10 (3, 50)	**<0.001** ^b^
	Count (%)	Count (%)	Count (%)	
Career status				**<0.001** ^c^
Active	1,270 (94.5)	1,027 (96.3)	224 (87.5)	
Inactive^2^	74 (5.5)	40 (3.7)	32 (12.5)	
Retired	47 (3.5)	24 (2.2)	23 (9.0)	
Health-related exit	25 (1.9)	13 (1.2)	10 (3.9)	
Career change	9 (0.7)	6 (0.6)	*	
Department/Type				**0.028** ^c^
Career	956 (71.1)	741 (69.4)	200 (78.1)	
Career & volunteer	242 (18.0)	197 (18.5)	39 (15.2)	
Volunteer	104 (7.7)	91 (8.5)	13 (5.1)	
Other^3^	42 (3.1)	38 (3.6)	*	
Most recent rank^2^				**<0.001** ^c^
Firefighter	850 (63.2)	709 (66.4)	129 (50.4)	
Paramedic/EMT^4^	486 (36.2)	390 (36.6)	89 (34.8)	
Company/Station officer	279 (20.8)	205 (19.2)	70 (27.3)	
Driver/Engineer	211 (15.7)	153 (14.3)	52 (20.3)	
Chief officer/Superintendent	97 (7.2)	75 (7.0)	22 (8.6)	
Master technician	13 (1.0)	10 (0.9)	*	
Other^5^	126 (9.4)	94 (8.8)	31 (12.1)	
Most recent assignment				0.473^c^
Engine	700 (52.1)	566 (53.0)	122 (47.7)	
Truck (Ladder/Aerial)	112 (8.3)	91 (8.5)	19 (7.4)	
EMS Unit (Ambulance)	105 (7.8)	79 (7.4)	23 (9.0)	
Special Operations / Rescue	56 (4.2)	49 (4.6)	7 (2.7)	
HazMat Unit	20 (1.5)	14 (1.3)	5 (2.0)	
Other^6^	346 (25.7)	266 (24.9)	80 (31.3)	

* = fewer than 5.Bold *p*-values indicate statistical significance.

1 Responses from respondents with or without a history of cancer and/or precancer may not add up to those of all respondents, as some respondents did not answer every question.

2 Questions in which participants could select more than one option.

3 “Other” reported departments included Wildland/Forest Service, Structural, Industrial, and Military.

4 EMT, Emergency Medical Technician.

5 “Other” ranks reported included [new] Recruit, Dispatcher, Shift Coordinator, Training Centre Coordinator, Training Officer, Qualified Communication Officer, Fire/Arson/Prevention/K-9 Investigator (working with trained dog), EMT/Fire Service Instructor, Medical/First Responder, Helicopter Rescue Firefighter Paramedic, Aquatic Rescue, Hazardous Materials Technician, Lieutenant, Deputy/Fire Marshall, and Battalion Chief.

6 “Other” recent assignments included Training, Public Information Officer, Law Enforcement Investigator, Fire Marshall/Code Enforcement, Fire Chief, Battalion Chief, Homeland Security, and Airport Fire Station/Aircraft Rescue Firefighting.

a *T*-test.

b Wilcoxon rank-sum test.

c Chi-square test.

Although the majority of respondents identified themselves as a firefighter, those with cancer were significantly more likely to be a Company or Station Officer, Driver or Engineer, or a Chief Officer or Superintendent (*p* < 0.001) (Table 2). There were no differences found between those with and without cancer in terms of most recent assignment, with 52.1% of all respondents being assigned to an engine, followed by truck (8.3%), and Emergency Medical Services (EMS) unit (7.8%).

A total of 372 respondents (27.7%) reported having taken a leave of absence; 30.1% among those with cancer and 27.1% among those without cancer. The average number of months of leave among those with cancer was higher than among those without cancer. Nearly three-quarters of respondents indicated that they have “presumptive coverage” workers’ compensation without having to prove their condition was caused by their work: A lower proportion of respondents with cancer reported having presumptive coverage than those without cancer.

A significantly higher proportion of respondents with cancer reported tobacco use as compared to those without cancer (*p* = 0.005) (Table 3). They also reported a longer period of time since starting tobacco use (*p* < 0.001), more years since stopping use (*p* = 0.039), and a significantly higher number of uses per day (*p* = 0.035). There was no difference between groups regarding those who smoked and those who used a smokeless tobacco product. There was no statistical difference in alcohol consumption between the female firefighters who had cancer versus those who did not; 91.2 percent of the sample reported alcohol consumption. A significantly higher proportion of respondents with cancer reported more years since starting alcohol consumption (*p* < 0.001), but there were no differences in years since stopping or the number of consumptions per week.

**Table 3:** Cancer risk factors of female firefighter survey respondents, by presence and absence of cancer.

Domain of inquiry	All respondents^1^ (*n* = 1,344)	Respondents without cancer (*n* = 1,067)	Respondents with cancer (*n* = 256)	Comparison
	Count (%)	Count (%)	Count (%)	*p*-value
Tobacco use^2^^,^^3^	347 (25.8)	258 (24.2)	84 (32.8)	**0.005** ^a^
Smoke	319 (23.7)	234 (21.9)	80 (31.3)	0.428^a^
Smokeless	36 (2.7)	29 (2.7)	7 (2.7)	
Alcohol consumption^3^	1,226 (91.2)	970 (90.9)	239 (93.4)	0.210^a^
Hormone use^2^	926 (68.9)	729 (68.3)	185 (72.3)	0.220^a^
Estrogen	560 (41.7)	435 (40.8)	118 (46.1)	0.076^a^
Progesterone/Progestin	558 (41.5)	448 (42.0)	106 (41.4)	
Testosterone	53 (3.9)	34 (3.2)	19 (7.4)	
Human growth hormone	12 (0.9)	10 (0.9)	*	
Androgen	8 (0.6)	6 (0.6)	*	
Glucocorticoid	*	*	0	
Other^4^	133 (9.9)	100 (9.4)	29 (11.3)	
Full-term pregnancy and delivery	609 (45.3)	456 (42.7)	143 (55.9)	**0.005** ^a^
Breastfeeding	536 (39.9)	407 (38.1)	122 (47.7)	0.201^a^
Family history of cancer				0.088^a^
Mother	277 (20.6)	222 (20.8)	55 (21.5)	
Father	246 (18.3)	196 (18.4)	50 (19.5)	
Sister	50 (3.7)	35 (3.3)	15 (5.9)	
Brother	34 (2.5)	22 (2.1)	12 (4.7)	
	Mean (SD) Median (Q1, Q3)	Mean (SD) Median (Q1, Q3)	Mean (SD) Median (Q1, Q3)	
Years since starting tobacco	24.3 (10.9)	22.9 (10.7)	28.7 (10.6)	**<0.001** ^b^
Years since stopping tobacco	16.8 (11.0)	16.1 (10.6)	19.1 (11.7)	**0.039** ^b^
# Tobacco uses per day	6 (3, 10)	5 (2,10)	7 (5,15)	**0.035** ^c^
Years since starting alcohol	21.9 (10.3)	20.7 (10.0)	26.9 (9.7)	**<0.001** ^b^
Years since stopping alcohol	1 (0.5, 9)	1 (0.5, 8)	4 (1, 12)	0.089^c^
# Alcohol consumptions/week	3 (1, 5)	3 (1, 5)	3 (1, 5)	0.575^c^

* = fewer than 5.Bold *p*-values indicate statistical significance.

1 Responses from respondents with or without a history of cancer and/or precancer may not add up to those of all respondents, as some respondents did not answer every question.

2 Questions in which participants could select more than one option.

3 Tobacco use and alcohol consumption are “in lifetime.”

4 “Other” hormones reported included bioidenticals, hormones for IVF/fertility treatments, Fibristal for treatment of fibroids, DHEA, and contraceptives/IUD.

a Chi-square test.

b *T*-test.

c Wilcoxon rank-sum test.

Hormone use was reported by 68.9% of respondents, with no differences between those with and without cancer (Table 3). Over 40% of the sample reported using estrogen and/or using progesterone, with less than 5% using other types of hormones. A significantly higher proportion of respondents with cancer reported having had a full-term pregnancy and delivery (*p* = 0.005), while there were no differences in terms of having breastfed. There were no differences in family history of cancer between the two groups.

### Social, psychological, and physical perspectives of working in the fire service

Fifteen respondents reported that firefighting is associated with many stressors that impact overall mental health. For instance, they reported struggling with anxiety, psychological distress, and post-traumatic stress disorder as a result of witnessing devastating human suffering and loss, such as responding to individuals who self-harm. Thirty participants highlighted challenges within the fire service that are unique to women, including: (a) being the only woman in the department; (b) feeling pressure to perform the same tasks as men; (c) bullying or hazing; (d) the challenges of returning to work after maternity leave; and, (e) adverse biological outcomes (e.g., issues with fertility). One participant explained,

*over my career it has been challenging, as a female, to do what is right and ride the fine line of being accepted and being ridiculed or written off because I am female. Ideas on safety are generally not accepted if they come from a female versus a male, in my experience.*

Respondents described that being the only woman resulted in a sense of isolation and thus they felt compelled to perform riskier, more physically demanding tasks in order to prove that they were as competent as their male counterparts. Some also noted a culture of bullying and hazing from their male colleagues, especially when they were the only women in the department. Additionally, respondents explained that returning to their job following a maternity leave was physically demanding. Lastly, they felt that increased exposure to toxins likely had an impact on their fertility or harmed their fetuses.

Being the only woman in the department sometimes meant a lack of access to basic requirements, such as a separate locker room. One firefighter described,

*I feel like my department has not done everything it can to protect us. For years I was at a station under construction with only one bathroom where I would have to hold my urine because other [firefighters] were using the bathroom. I did not (and still do not) have adequate female locker facilities at all stations.*

Those commenting on health and safety measures at their departments indicated that their units were proactive with health and safety education and ensured firefighters had access to appropriate and clean personal protective equipment. One respondent described the measures in their department: “A few years ago our department bought a unit for members to properly decontaminate after a fire. We shower, then ride [a] bike in the decontamination unit for 20 min at 26 degrees and shower again.” Other respondents noted that their departments were implementing notable changes toward improving health and safety for the firefighters, including improvements to: decontamination procedures, protective gear, health and safety standards, and addressing mental health. They expressed that they appreciated and supported the shifting focus toward implementing health and safety standards within fire departments.

When asked whether there was anything they would like to share about their career experience with the fire service, 94 respondents described occupational hazards they encountered routinely throughout their employment including: exposure to toxins (*n* = 54), inadequate equipment (*n* = 33), overscheduling (*n* = 4), and the overall culture of their fire service (*n* = 9). Overwhelmingly, respondents cited frequent exposure to toxins (e.g., airborne, water-borne, on their skin), including toxins from smoke inhalation, cleaning supplies, fire truck diesel fuel and exhaust, and other chemicals. One participant described, “we have a flashover simulator that is used for training. I understand it is important to learn about fire behavior, but do not believe it is necessary to continue using this training again and again if it can contribute to cancer.” Another stated, “I’ve been to fires where fertilizer has been part of what’s burning but no one ever told us that was dangerous or to protect ourselves, it was do your job or quit.” Others also described responding to fires such as these – in which they were unaware of what was burning – which increased their risk of exposure to dangerous toxins. Another respondent explained the common exposures resulting from fire trucks:

*I feel that regular or daily exposure to diesel exhaust from fire trucks play a large role in our toxic exposure. While I find it difficult to look back and count specific events that had toxic exposure, because there are so many, I personally feel that diesel exhaust exposure is highly toxic and crews are exposed daily.*

With regard to inadequate equipment, many participants explained that: (a) they did not have access to appropriate personal protective equipment; (b) the equipment they did have access to did not fit (as it was intended for male firefighters); or (c) the equipment was not decontaminated after use. Many respondents reported not having access to a self-contained breathing apparatus or protective mask. As one individual expressed, “[I had] ill-fitting equipment: Even wearing a small [breathing apparatus] mask will not pass [a] fit test; [my] current helmet … is the subject of lawsuits in [the United States because it] causes neck fatigue/pain.” Some participants noted that they had been scheduled for extensively long shifts, thus increasing their fatigue and stress. Several respondents explained that their overall work environment felt ‘toxic’ in that they felt their department did not take the health and safety of their firefighters seriously enough. As one individual explained,

*[There is] contradictory behaviour … towards health and safety. [The fire service] promote [s] measures and programs to the media but [exhibits] harassment and/or threatening behaviour towards individuals in our department who require shifts off due to physical injury or sickness (even with medical documentation) or that need a shift or two off due to mental stress. As a result, individuals are coming to work when they should not be (injury or sickness), due to fear of attendance management programs. [This] creates a dangerous atmosphere.*

A number of respondents indicated positive experiences with the fire service, stating that they found their career rewarding (*n* = 19), their departments had safety measures in place (*n* = 7), or that their departments were making encouraging changes to create safer workplace environments (*n* = 14). Some described that they enjoyed being able to give back to their communities through their service.

### Clinicobiological perspectives of cancer and precancer experiences

A total of 256 respondents experienced a diagnosis of cancer. The average age at the time of diagnosis, reported by 206 respondents, was 39.0 years (SD 10.1), with ages ranging from 15.6 to 65.7 years. The major types of cancer reported included breast, cervical, melanoma, basel cell/skin, uterine, colorectal, thyroid, and ovarian (Table 4).

**Table 4:** Cancers experienced by female firefighter survey respondents, by precancer or cancer diagnosis, and by mode of detection.

Type of cancer	All cases^1^ (*n* = 256)	Precancer (*n* = 166)	Invasive cancer (*n* = 117)		Mode of detection			
				Total reported	Self-detected	Routine exam	Cancer screen	Other^3^
	Count (%)	Count	Count	Count	Count	Count	Count	
Breast	65 (25.4)	21	37	61	23	9	21	8
Cervical	54 (21.1)	24	5	48	5	17	24	*
Melanoma	53 (20.7)	34	22	50	26	10	10	*
Basal cell / Skin	42 (16.4)	16	5	38	17	14	6	*
Uterine	38 (14.8)	31	12	31	10	15	6	0
Colorectal	16 (6.3)	11	7	15	6	5	*	*
Thyroid	15 (5.9)	6	8	14	5	*	5	*
Ovarian	13 (5.1)	6	*	13	6	5	*	0
Leukemia	*	*	*	*	*	0	0	0
Bladder	*	*	*	*	*	0	0	0
Oropharyngeal	*	0	*	*	0	0	*	*
Brain/CNS	*	*	*	*	*	0	0	0
Lung/Bronchus	*	*	*	*	0	*	0	0
Non-Hodgkin lymphoma	*	0	0	*	*	0	0	0
Kidney / Renal pelvis		*	*	0	0	0	0	0
Other^2^	5 (2.0)	*	*	*	*	*	0	0

* = fewer than 5.

1 Individual incipient (precancer) and invasive cancer numbers may not sum to the combined total due to participants responses.

2 Other type of neoplastic processes included: cholangiocarcinoma, meningioma, MGUS – Monoclonal gammopathy of undetermined significance, vulvar.

3 Other modes of detection included: biopsy on breast reduction, detected during exam for other issues, ENT (ear, nose, and throat specialist) detected.

The timing of cancer detection was reported by 93.8% of these respondents; 13.7% of cases occurred prior to fire service, 73.4% during active fire service, and 6.6% following fire service employment. The means of detecting the cancer was reported by 89.1%; of these, 42.1% were detected when the respondent sought medical attention after noticing concerning signs or symptoms, 29.8% during a routine health screen, 28.1% during a specific cancer screen, and 6.6% were detected by other means, such as biopsy during breast reduction. At the time of the study, the average number of years since detection was 7.0 (SD 7.2) for the full sample. For those whose cancer was detected after leaving the fire service (*n* = 17), the average number of years between leaving the service and detection was 4.6 years (SD 5.5).

Of the 256 respondents with cancer, 44.5% reported the stage and 30.9% reported the histopathological grade of their cancer. Surgery was the predominant treatment, followed distantly by chemotherapy, radiotherapy, and observation. Further details are presented in Table 5.

**Table 5:** Cancers experienced by female firefighter survey respondents, with invasive cancer or incipient cancer, by stage, grade, and treatment type.

Invasive cancer																
			Stage	Grade					Treatment^1^				
		0	I	II	III	IV	Undetermined	Well Differentiated	Moderately Differentiated	Poorly Differentiated	Un-differentiated	Surgery	Chemo-therapy	Radiotherapy	Observation	Other^2^
Type of cancer	*n*	Count	Count	Count	Count	Count	Count	Count	Count	Count	Count	Count	Count	Count	Count	Count
Breast	37	9	13	7	6	0	*	*	11	7	*	34	15	18	*	5
Cervical	5	*	*	0	0	0	*	*	0	0	0	*	0	0	*	0
Melanoma	22	6	5	*	*	0	5	*	*	0	0	22	*	*	0	*
Basal cell/Skin	5	0	*	0	0	0	*	0	0	0	0	5	0	0	0	0
Uterine	12	*	*	0	*	0	*	*	*	0	0	11	*	*	*	*
Colorectal	7	*	*	0	*	0	*	0	*	0	0	6	*	*	*	0
Thyroid	8	0	*	*	*	0	0	*	*	0	0	8	0	*	0	0
Ovarian	*	0	0	0	*	0	*	0	0	0	0	*	*	0	0	0
Other	7	*	*	*	0	0	5	*	*	0	*	5	*	*	0	0
**Incipient cancer**																
		Stage					Grade					Treatment^1^				
Type of cancer	*n*	Count	Count	Count	Count	Count	Count	Count	Count	Count	Count	Count	Count	Count	Count	Count
Breast	21	4	*	*	*	0	*	*	*	*	0	16	7	7	*	*
Cervical	24	7	*	*	*	0	*	*	0	0	*	14	*	*	*	*
Melanoma	34	7	*	*	0	0	5	*	*	*	0	29	*	0	*	*
Basal cell/Skin	15	1	*	*	*	0	*	0	*	0	0	12	0	*	*	*
Uterine	31	3	*	*	0	0	*	*	*	*	0	21	*	*	7	*
Colorectal	11	3	0	0	*	0	*	0	0	0	0	6	*	0	*	*
Thyroid	6	0	*	*	0	0	*	*	*	0	0	*	0	*	*	0
Ovarian	6	1	*	0	*	0	*	*	0	0	0	6	*	0	0	0
Other	8	2	*	*	0	0	*	*	0	*	0	5	*	*	*	0

* = fewer than 5.

1 Questions in which participants could select more than one option.

2 Other treatments reported by respondents included estrogen blocking, IUD placement with progesterone, cryotherapy, laser removal, LEAP – biologically enhanced particle therapy, immunotherapy, acupuncture, herbs, diet, and energy work.

### Social, psychological, and physical perspectives of cancer-related experiences

Of the 256 participants who experienced cancer, 45.7% reported not having experienced any cancer-related challenges. Several participants (*n* = 21) explained that their condition was precancerous or that their cancer had little-to-no impact on their daily lives. They noted that either they did not develop invasive cancer or that their conditions were detected early and treated quickly. One participant described, “through regular physicals and pap smears the precancerous cells were discovered – once before employment and the second time after employment. Easily treated and minimal after effects.” Although the respondents explained that there was no impact on their quality of life, they did acknowledge that waiting for their test results caused heightened stress.

Many respondents described challenges resulting from their cancer or associated treatments: 33.2% reported psychosocial, 18.8% financial, 6.6% physical, and 6.3% spiritual challenges. Twenty-seven respondents shared negative health outcomes resulting from their cancer medications, treatments, or surgeries.

Physical challenges and limitations included pain, feeling ill, recovering from surgery, and changes in energy levels, appearance (e.g., scarring, weight loss), or anatomy (e.g., removal of section of their colon). Others expressed that their physical activity levels had decreased as well as their ability to engage in activities of daily living. As one respondent described,

*[cancer] turned [my quality of life] upside down! Three years after diagnosis, I am ‘cancer free’ but still have treatments and surgeries. People think because I look ‘better’ on the outside I should be done and back on the truck running calls. They do not get that the joint pain continues and the [chemotherapy], radiation, mastectomy, and reconstruction have left me weak and scarred.*

Twenty-two respondents highlighted psychological challenges as a result of their cancer. Many described that their stress initiated from constant worry that their cancer would return or worsen. One respondent explained, “[I felt] stress waiting for test results, and now that I’ve had skin cancers removed, I’m paranoid of finding more or worse ones.” Some described that although they had survived their cancer, they were mentally not able to accept that they had recovered: “it’s a big deal to go through cancer. All is well, but it’s still a process getting through it all and not thinking you do not have cancer every single day.”

Social challenges were described (*n* = 7) relating to the impact cancer had on the respondent’s personal relationships. The cancer experience created stress on family members and, for some, resulted in ruptured relationships. Some expressed that they lacked a strong social support system, and that going through cancer made them feel further isolated.

Five respondents also noted financial strain related to their cancer experience, such as having to take an early retirement, or medical or restorative procedure costs. One person explained:

*Being a contractor, I didn’t have any income or disability during the year and a half treatment. The only option I had left was to live in a van to finish out treatment and I’m so grateful I could afford that. … I have had to go back to work for financial reasons and cosmetically my surgeries are not done which has left me with social anxiety and self-esteem issues. I just want to help raise awareness for prevention. And help others be aware of how many resources are needed for someone with cancer so they may be completely supported.*

Seven participants emphasized the positive impact cancer had on their lives. They made changes to their diet (e.g., eating healthy foods), routine (e.g., practicing sun protection and attending cancer screening regularly), and mindset (e.g., positive outlook). A participant stated, “It sucks. I hate having to live with the constant reminder of [cancer]; however, I am not allowing it to determine my progress. It’s just something I have and I am making adjustments to my lifestyle to help reduce reoccurrence.”

### Social and psychological perspectives of reporting cancer to employers

The leading concerns about reporting a cancer experience to their employer was the desire to keep health information private (11.3%), followed by a feeling of vulnerability (7.4%), being perceived as weak (7.0%), experiencing discrimination from superiors (5.5%), and losing credibility (5.1%). Concerns reported by fewer than 5% of respondents included embarrassment, experiencing discrimination from coworkers, and experiencing harassment from coworkers.

Forty-five participants further described barriers to reporting their cancer to their workplace, these included: lack of support from their employer or insurer (*n* = 25); increased workplace stress (*n* = 17); and the prospect of having to engage in ongoing litigation with the fire service (*n* = 3). The lack of support felt by respondents included feeling that the seriousness of their condition was misunderstood or minimized by their employers, the need to use sick time for treatment or surgery, and the fear of job loss. One respondent described their experience, stating:

*There is lack of support in using sick time benefits for doctor’s appointments and procedures. [My employer] ask [s] too many personal questions that are not relevant when accessing sick time benefits. This lack of support adds stress when I’m already dealing with something emotionally taxing. I worry about having to deal with my employer when I need to take time off work for a serious health concern.*

Having to take sick time or vacation days for appointments related to their cancer was met with resistance from some employers regardless of the severity of their condition. Another participant expressed that “cancer is so prevalent in the fire service now that our department has become numb toward it.” And another stated that they felt concerned about reporting their cancer because they “did not want there to be any reason for my job to be in jeopardy.”

Many respondents described the difficulty with securing medical coverage and workers’ compensation for their cancers. As one participant explained:

*I spend a lot of days off going to the [doctor] and getting treatments. These appointments consume me and it feels like my entire life has been affected. Yet the only compensation are the days that I miss work for it, but my whole life is affected.*

Respondents explained that the prospect of having to engage in ongoing litigation with their employer in order to be awarded workers’ compensation benefits was another source of stress. One participant described that they have been trying to settle their case for over 11 years, while another explained that they are experiencing “frustration that workers’ [compensation] is denying my cancer claim, even with presumptive laws for my cancer. All [city name] Firefighters are being denied by the city.”

### Social and psychological perspectives of cancer and firefighting

A number of respondents (*n* = 24) indicated that they developed cancer during or following their career in the fire service, and 40 respondents described added challenges with insurance and presumptive coverage for their cancers. Respondents described that that they developed various forms of cancer due to workplace hazards, and that cancer was also prevalent among their colleagues. Some respondents noted that cancers specific to women (e.g., ovarian) were not covered under presumptive disability but those specific to men (e.g., prostate) were, further indicating systemic barriers to supporting women in the fire service.

Presumptive coverage was seen to vary depending on the geographical location of the respondent; however, the challenges reported remained consistent across respondents. For instance, an individual in Virginia stated

*presumption in Virginia (USA) is highly limiting. Minor sprains and strains are covered but it’s horrifying how difficult [it is] getting approval for heart and lung [conditions] or cancer coverage. Only a short list of cancers is covered and of those they try to force patients to identify the exact single exposure to a specific carcinogen that caused their cancer.*

While another participant in Texas expressed,

*Workers’ [compensation] is currently denying my cancer claim for Stage III malignant melanoma in Texas even though the law has presumptive new laws for malignant melanoma. All claims are automatically denied. We are forced to go to court in the [city name] Fire Department.*

These respondents have highlighted that not only did they develop cancer during or following their career as a result of their workplace exposures, they were not able to obtain any support or workers’ compensation to assist them during their treatment and recovery.

Fourteen respondents described the impact that cancer had on their job. Seven of these respondents explained that they had to take a lot of time off work for recovery, others noted that it was difficult for them to return to their job due to physical limitations, and some described having to transition from operational firefighting to less physically demanding roles, such as trainer or in office.

## Discussion

Female firefighters are consistently underrepresented in the work-related illness evidence base as their numbers have been few. In order to appropriately support women in the fire service, more epidemiological evidence is required, ideally by establishing population-based cancer registries. This study describes the circumstances around a cancer or precancer diagnosis among female firefighters, including types of cancers reported, nature of work-related exposures, personal lifestyle factors, means of clinical presentation and diagnosis, therapeutic interventions and outcomes, and the impact that such a diagnosis has on the quality of life of a woman in the fire service.

Cancer is a leading cause of death in North America (25), and while firefighters tend to be healthy, physically fit, and less prone to chronic illness (26), they are still subject to the same basic risk factors for cancer as the general population. However, this risk is compounded by the cumulative toxicity arising from repeated and essentially unavoidable carcinogenic exposures in the line-of-duty (27). In a study of nearly 30,000 US career firefighters, Daniels and colleagues found that they generally developed less chronic disease but conversely acquired cancer 9% more frequently and were 14% more likely to die from this illness than the general population (26, 28).

Firefighters have been encouraged to wear their bunker gear and breathing apparatus appropriately in the belief that this equipment will protect them from the toxic smoke and fumes generated by a fire. However, because of the small molecular size, combined with the tissue and technical-fabric permeability of gaseous-phase fire-generated carcinogens, these compounds pass with facility through most modern turnout suits and from there are absorbed into the body via inhalation, ingestion, or directly through the skin (29, 30). Firefighters anecdotally but universally report that after a fire, despite the rigorous use of personal protective equipment and meticulous decontamination, the odor of smoke and burning materials linger with them for days, evidence that their bodies are off-gassing carcinogens long after knockdown and overhaul of the fire (KRK, personal communication, September 6, 2022). Recent research has confirmed that improved personal protective equipment is needed, as current decontamination protocol for washing following a firefighting event is insufficient to protect firefighters from carcinogenic exposures, such as to polycyclic aromatic hydrocarbons (31).

Given the carcinogenicity of firefighting, the question arises as to which types of cancers firefighters are most vulnerable. This can be a contentious political issue, as some governmental jurisdictions recognize one variety of cancer, but will not compensate for another. Many studies of firefighters and other individuals subject to similar toxic exposures have demonstrated that carcinogenic compounds or their metabolites can be isolated from a variety of biologic fluids and elimination products, including blood, urine, stool, breast milk, and semen, among others (32–38). This widespread systemic uptake, distribution, and subsequent excretion of toxins highlights the fact that, in the course of their duties and over time, the bodies of firefighters become repeatedly saturated with innumerable carcinogens (6). This places them at risk of acquiring any type of cancer involving any organ system, since all organ systems are exposed either directly through environmental contact or via the circulatory system.

So constitutionally permeating is the effect of this toxicity, that it even transmits vertically to the offspring of firefighters. Research has shown that the progeny of both male and female firefighters are subject to such untoward effects as an increased rate of birth defects (38), low birth weight at parturition, and elevated rates of spontaneous miscarriage (21). The latter findings implicate the existence of a penetrating genotoxic mechanism that acts even upon germ cells in the reproductive glands of firefighters.

As this study features a set of 256 cases of cancer or preinvasive cancer reported by female firefighters, the question arises as to what types of cancers have been disclosed and how the frequency, biological nature, and clinical characteristics of these cancers compare to those experienced among women in the general public. Similar to other accounts (9, 11, 12, 28), this study observed a wide variety of different types of cancers. In order of descending frequency, these were comprised of breast, cervical, melanoma, skin, uterine, colorectal, thyroid, and ovarian cancer, among others. These frequencies are different from what is seen in the general population, as this study shows higher proportions of cervical, melanoma, and ovarian cancer cases than would have been expected in the general public.

Breast cancer was the most common, which aligns with what is seen in the general population, as breast cancer is the most frequent cancer occurring in women (2, 3). Most of the study cases were discovered either by the firefighters themselves or on screening mammography, emphasizing the importance of awareness. Similar to the general population, most of the tumors were found in early clinical stages and were histopathologically well- or moderately well-differentiated (16). Surgery is the mainstay of treatment for breast cancer, thus most of the firefighters reported undergoing surgery, with some cases followed by radiotherapy and chemotherapy. Importantly, the clinical and biological behavior of the breast cancers seen in women in the fire service did not seem more aggressive than that of the general population, except for the notably younger average age of the firefighters. Breast cancer in the public mainly occurs at a median age of 62 years (39), whereas the mean age of firefighters with cancer responding to this study was 39 years.

The second most frequently reported type of malignancy in this study was cancer of the cervix. This finding is different from the public, where it is typically not among the top 10 most frequent cancers among women (2, 3). Most of the cervical cancer cases disclosed were pre-invasive lesions discovered by routine medical examination or on dedicated cancer screening, and were effectively managed with surgery, which aligns with usual clinical care. Since cervical cancer is most often diagnosed in women between the ages of 35 and 44 years, with the mean age being 50 (40), it is not surprising that so many cases appeared in this series. Previous studies have also found an elevated risk of cervical cancer among female firefighters (9, 41). As the clinical course of cancer of the cervix can be aggressive if left unchecked, awareness and early intervention can significantly decrease morbidity from this illness.

Melanoma was the third most common form of cancer disclosed in this study, followed by the typically benign basal cell skin cancer. Melanoma is generally the 6^th^ leading type of cancer found among women in the public (3), with age-standardized rates highest in North America, parts of Europe, and Australia (42). Melanoma has the potential to be progressive and fatal, whereas a skin tumor such as basal cell is common, benign, and easily treated (43). Both conditions include exposure to ultraviolet light and Caucasian ethnicity as risk factors (44), and have been associated with the carcinogens encountered during firefighting (45). Participants in this study may have been at a higher genetic risk than the general population, as they were predominantly Caucasian. Most of the melanoma cases in this series were detected either by the firefighters themselves or on routine screening, as this variety of cancer is often visible on the skin as an irregular, enlarging, dark or multicolored shape. The melanoma cases were found in an early clinical stage and managed by surgery, which is the treatment of choice. Cancer mechanism-wise, the skin is the largest organ of the body, is a very metabolically active tissue, and covers a wide surface area. As fire-generated carcinogens penetrate bunker gear and are absorbed into hot, moist skin, this would provide, in contrast to solar-derived ultraviolet exposure, a rationale for the higher melanoma rates consistently reported among firefighters (8, 11, 12, 46). Fortunately, the melanoma cases in this study did not appear to be more aggressive than those observed in the public. With increasing awareness among firefighters and their healthcare providers, early diagnosis and intervention will allow for the best clinical outcome.

In the present series, uterine and thyroid were the 5th and 7th most common cancers, respectively. They were generally low-grade lesions found in early clinical stage by self-detection or on medical screening and treated with standard therapy. Their clinical and biological behavior patterns were not noticeably different from these types of cancers when they occur in the public.

Lung cancer is the second leading type of cancer among women in the general population (2, 3), and is one of the most fatal types of malignancies (47). Lung cancer rates have been reported in the fire service by several sources, with varying conclusions about its association with firefighting (7, 10, 26, 27, 46, 48). The present study did not find a high proportion of lung cancer cases among female firefighters. This may be due to the relatively young overall average age of all the participants, which was 41-years, as most people diagnosed with lung cancer are 65-years-of-age or older (47). Because lung cancer is often treatment-resistant and diagnosed in an advanced stage and follows a rapidly fatal clinical course, an alternative explanation may be that female firefighters with lung cancer were either already deceased or were too ill to participate.

Colorectal cancer is the third leading type of cancer occurring among women (49), and has been found to be elevated among firefighters in previous studies (7, 48, 50, 51). However, similar to the arguments for lung cancer above, there were few cases of colorectal cancer disclosed in this survey. The average age of the participants at time of diagnosis was 39-years, whereas the average age of onset for colorectal cancer among women in the public is 72 (52). The invasive colorectal cancer cases reported here were discovered primarily by self-detection or during routine examination, and were found mainly in early clinical stages, with surgery as the primary form of medical intervention.

The present report features 13 cases of ovarian cancer or precancer out of a total 256 of cases, making it the 8th most common malignancy in this study, whereas it is much less frequent among women in the public (2, 3). This suggests that ovarian cancer may be more prevalent in the fire service than previously recognized. This finding may not be surprising, as although ovarian and testicular cancer have different clinical courses, ovaries are derived from the same endodermal tissue as testicles, and testicular cancer is commonly elevated among male firefighters (7, 8, 11). Left untreated, ovarian cancer is typically an aggressive disease with a high mortality rate, and it is not often included in cancer presumption legislation for female firefighters. The fire service should be aware that the relative frequency of ovarian cancer may be higher among female firefighters than the general population so that steps can be taken to improve awareness, surveillance, support, and early diagnosis.

Most of the reported cancer cases in this study were detected while the firefighter was actively employed in the fire service, which suggests that cancers linked to firefighting may come sooner than what is generally believed. Those female firefighters with cancer were found to be older, on average, than those without cancer, as age is the most import risk factor for acquiring cancer. Many characteristics of this female firefighter cancer sample are consistent with being older. For example, as compared to those without cancer, these women reported more years in the fire service, more exposures to fires and toxins, a larger proportion in higher positions within the fire service, and a larger proportion of women no longer in the fire service whether due to retirement, health problems, or career change. Female firefighters with cancer also reported longer periods of tobacco use and alcohol consumption, higher tobacco usage, and more years since stopping tobacco use than those without cancer. Tobacco use is an important indicator of stress and mental health challenges (53), as well as a risk factor for cancer (3). Current or previous tobacco use was reported by 32.8% of female firefighters with cancer and 24.2% of those without cancer, as compared to only 10–12% of the general public (54, 55). While higher numbers of completed pregnancies has been regarded as protective against some types of reproductive cancers (56), more of the older female firefighters with cancer reported completed pregnancies than the younger women without cancer, due to opportunity and stage of life. No differences were found between the two groups regarding hormone use, breast feeding, or family history of cancer.

Beyond exposure to chemical carcinogens, emotional stress is a well-known risk factor for cancer (57), presumably by weakening the immune system through altering hormone levels, and negatively influencing personal lifestyle choices such as diet, exercise, and the consumption of tobacco, alcohol, and illicit drugs. Some female firefighters report having to face a male-dominated workplace culture that at times features elements of discrimination or harassment (58, 59). Female firefighters in this study reported such feelings as a sense of isolation when they were the only woman in the department, feeling pressure to perform the same strenuous physical tasks as men, and experiencing bullying or hazing. Other challenges they experienced include concerns around the impact of toxins on their fertility or their unborn fetuses, and the physical demands of returning to work following a maternity leave. Anecdotally, firefighters have expressed concern about exposing their families to toxins they have carried home from work, or risking the well-being of their infants through contaminated breastmilk (KRK, personal communication, September 6, 2022).

Other concerns centered around fire stations not being equipped to provide adequate accommodations for women, such as locker facilities or bathrooms, or the availability of properly fitting personal protective equipment (60). Nonetheless, a few women reported on the positive aspects of their work, including a sense of personal reward derived from the challenge of firefighting as well as the opportunity of contributing to their communities, and a sense of reassurance that their departments were working to create safer workplaces.

Those firefighters with cancer or precancer who were diagnosed and treated quickly, reported experiencing little negative impact from their condition. Negative impingements that were reported by female firefighters with cancer included physical challenges and limitations in the form of pain, illness, decreased energy, and disfiguring cosmetic changes resulting from procedures such as mastectomy or hysterectomy. Stressful psychological challenges were also reported through such instances as anxiety while waiting for test results, or worrying about worsening or recurring cancers. Social challenges were encountered in terms of perceived impact on a firefighters’ employability, strain on relationships with others, stress on family members, and lack of support.

An overhanging concern for a firefighter when confronted with a diagnosis of cancer is the question of whether or not they will be eligible for financial compensation. When such firefighters are denied workers’ compensation, it not only places them under financial strain, but also denies a formal public recognition of their service (61). De Boer and colleagues found that, in general, cancer survivors experienced more medical costs and reduced earnings than those who had not experienced cancer (62). Female firefighters experiencing cancer in this study reported financial difficulties resulting from medical bills, lack of disability insurance, and being required to take early retirement. A larger proportion of female firefighters without cancer reported that their employee health insurance included presumptive coverage for specified injuries or diseases presumed to be work-related than those who actually experienced a diagnosis of cancer. It is possible that those who had experienced cancer and then attempted to access resources had a better understanding of what their presumptive coverage actually provided. They reported having to fight for workers’ compensation benefits even for cancers falling under the presumptive coverage, and having persistent outstanding reimbursement claims.

In this study, some female firefighters reported concerns about revealing their cancer diagnosis to their employer. They expressed the desire to keep health information private, and also expressed feeling vulnerable but not wanting to be perceived as weak. Fitness or ability to maintain employment is a known psychosocial issue among cancer survivors (63). Perceived barriers to the ability to work include discrimination and the perception that the employee can no longer perform the required tasks (64). A lack of understanding or empathy from employers was also reported, stemming from employer concerns with using sick time for medical appointments, employees having to use vacation time to move through their illness, as well as a fear of job loss. Women in this study reported challenges to returning to work after cancer treatment due to their own physical limitations, requiring workplace accommodations or transitioning to less demanding roles.

Positive impacts from experiencing cancer reported by female firefighters included choosing to make lifestyle improvements, practicing prevention and regular cancer screening, and cultivating a positive outlook on life. Furthermore, many cancer survivors feel inspired to start or participate in support programs to mentor others who are coping with a cancer diagnosis, as exemplified by the Firefighter Cancer Support Network^1^ in the US.

It is clear that the mechanisms or policies designed to support women in the fire service, both in general and when they face a cancer diagnosis, are currently insufficient. Some female fighters continue to experience discrimination in this male-dominated profession, and are resourced with insufficient facilities or gear. Furthermore, when firefighters are diagnosed with cancer, they need help. The fire service should anticipate the specific needs of women, from managing their illness and recovery, to their financial burden via workers’ compensation, and reintegration back to work. Firefighting agencies should anticipate that cancer among women firefighters will be a continually emerging problem. Smooth mechanisms must be in place to deal with this exigency, in terms of prevention, screening, and presumptive coverage.

The limitations of this study include selection bias, the use of self-reported information, and incomplete data. Selection bias, where women in the fire service who have experienced cancer, or work-related injury, may have been more likely to participate in this study than those who have not, may explain the differences in cancer frequency as compared to the general population, and the younger average age at diagnosis. Diagnostic bias is also possible, as firefighters may participate in cancer screening more so than the general population. Accuracy of the responses, particularly concerning cancer and precancer diagnoses, could not be verified since the participating firefighters’ medical records were not available for review. The present report could not capture all female firefighters who experienced a diagnosis of cancer during the study period, as some may not have been aware of the survey or those who were may have not been willing to share their experiences. Although patient access to online medical records continues to improve, some patients diagnosed with cancer choose not to look (65); many do not want to know or discuss the details of their illness because it involves a direct confrontation with their loss of wellbeing or even their mortality (66) (KRK, personal communication, September 6, 2022). Finally, as cancer is a debilitating and life-threatening illness, some women may have been too ill to participate or they may have succumbed to their cancer during the study period.

## Conclusion

Although much needed datasets concerning the health of female firefighters are beginning to appear in the literature, few studies address the specific details surrounding a diagnosis of cancer, including the individual clinical, social, psychological, physical, and personal struggles of a female firefighter negotiating such a devasting illness. Female firefighters, and the institutions that support them, should be aware that women in the fire service are vulnerable to a wide range of different types of work-related cancers, many of which are unique to women, and that these cancers may come earlier in life than those encountered by women in the general public. These findings are of concern, as many workers’ compensation programs regard only a few select types of cancers as being work-related. Therefore, many firefighters with less common cancers, or diagnostic timeframes that do not meet minimum exposure periods, are left ineligible for compensation benefits, and by extension, public recognition of their sacrifice in the line-of-duty. The observations reported here are important for continuing to raise awareness among female firefighters regarding their long-term occupational health risks; developing and evaluating health and wellness policies; designating supportive resources; and designing screening, surveillance, and prevention strategies to make the workplace safer for female firefighters. The information presented in this study will also inform policymakers with respect to the need for broadening cancer presumption legislation as it applies to female firefighters, and to encourage the ongoing development of firefighter cancer registries to facilitate research into the health and wellbeing of women in the fire service.

## Data availability statement

The datasets presented in this article are not readily available because of ethical confidentiality restrictions. Requests to access the datasets should be directed to the corresponding author.

## Ethics statement

This study was reviewed and approved by the University of British Columbia Children’s and Women’s Research Ethics Board (H18-03318). Written informed consent was obtained from all survey respondents for their participation in this study.

## Author contributions

KK, LG, and IP: conceptualization. KT: investigation. SP, SK, AZ, AP, and AW: data curation. KK, SP, SK, and KT: analysis and interpretation of data, writing original draft, and visualization. IP: supervision. KK, KT, SP, AZ, AP, AW, SK, LG, LT, and IP review and editing. All authors contributed to the article and approved the submitted version.

## Funding

This work was funded by City of Surrey Fire Department (grant number F19-00432).

## Conflict of interest

The authors declare that the research was conducted in the absence of any commercial or financial relationships that could be construed as a potential conflict of interest.

## Publisher’s note

All claims expressed in this article are solely those of the authors and do not necessarily represent those of their affiliated organizations, or those of the publisher, the editors and the reviewers. Any product that may be evaluated in this article, or claim that may be made by its manufacturer, is not guaranteed or endorsed by the publisher.

## Footnotes

^1^ https://firefightercancersupport.org/
